# Removal of hydrocarbons and heavy metals from petroleum water by modern green nanotechnology methods

**DOI:** 10.1038/s41598-023-32938-1

**Published:** 2023-04-06

**Authors:** Abderrhmane Bouafia, Souhaila Meneceur, Souheyla Chami, Salah Eddine Laouini, Henda Daoudi, Souheila Legmairi, Hamdi Ali Mohammed Mohammed, Narimene Aoun, Farid Menaa

**Affiliations:** 1 Department of Process Engineering, Faculty of Technology, University of El Oued, 39000 El-Oued, Algeria; 2 Laboratory of Biotechnology Biomaterial and Condensed Matter, Faculty of Technology, University of El Oued, 39000 El-Oued, Algeria; 3 Laboratory of Polymers Treatment & Forming, Faculty of Technology, M’Hamed Bougara University, 35000 Boumerdes, Algeria; 4 Laboratory of Bioresources, Integrative Biology and Exploiting, Biotechnology Higher Institute, Monastir University, 5000 Monastir, Tunisia; 5 Department of Chemistry, Faculty of Exact Sciences and Informatics, University of Jijel, 18000 Jijel, Algeria; 6 Department of Nanomedicine and Advanced Technologies, CIC-Fluorotronics, Inc., San Diego, CA 92037 USA

**Keywords:** Environmental biotechnology, Environmental chemistry

## Abstract

Considered heavy metals, such as As(III), Bi(II), Cd(II), Cr(VI), Mn(II), Mo(II), Ni(II), Pb(II), Sb(III), Se(-II), Zn(II), and contaminating chemical compounds (monocyclic aromatic hydrocarbons such as phenolic or polycyclic derivatives) in wastewater (petrochemical industries: oil and gas production plants) are currently a major concern in environmental toxicology due to their toxic effects on aquatic and terrestrial life. In order to maintain biodiversity, hydrosphere ecosystems, and people, it is crucial to remove these heavy metals and polluting chemical compounds from the watery environment. In this study, different Nanoparticles (α-Fe_2_O_3_, CuO, and ZnO) were synthesized by green synthesis method using *Portulaca oleracea* leaf extract and characterized by UV–Vis spectrophotometers, FTIR spectroscopy, X-Ray Diffraction (XRD), Scanning Electron Microscopy (SEM), Energy Dispersive Spectroscopy (EDS) techniques in order to investigate morphology, composition, and crystalline structure of NPs, these were then used as adsorbent for the removal of As(III), Bi(II), Cd(II), Cr(VI), Mn(II), Mo(II), Ni(II), Pb(II), Sb(III), Se(-II), and Zn(II) from wastewater, and removal efficiencies of were obtained 100% under optimal conditions.

## Introduction

The massive amount of water waste produced during the extraction of crude oil is known as produced water. It consists of oil well injection water mixed with formation water that is already present in the well. Produced water dissolves solids and suspended solids and contains a high oil concentration. This research is interested in treating the resulting water from the ALGERIA South oil company^1^.

In the oil industry, the growing demand for crude oil leads to very significant discharges causing irreversible and irreparable damage to nature and the environment. Among these discharges, we can cite the deposit waters accompanied by crude oil which are obtained at the level of the separator at the exit of oil wells, characterized essentially by high levels of hydrocarbons as well as by high levels of suspended solids and Heavy metals^2^. These metals are resistant to environmental degradation, difficult to metabolize, and have the potential to build up in the food chains of humans or ecosystems through ingestion^3^.

To fight effectively against these pollution problems, the consequences of which are varied, several solutions have been put forward by researchers in this field but require colossal means that the oil companies must take charge of. Among the solutions, it is to characterize, treat and valorize this deposit water by eliminating heavy metals and avoiding the formation of sloughs, a source of groundwater pollution^4^.

Currently, there are ways to treat water from the oil and gas industries with expensive raw materials, with a huge cost of producing treated water for possible reuse^5^. As an indication, these processes have certain limitations such as the inability to eliminate certain elements and the production of secondary waste requiring additional treatment. The goal of this project is to manufacture compounds of nanometric size, locally at a lower cost, and having high performance to eliminate waste of different types and sizes.

In today's world, researchers and scientists are increasingly interested in inorganic nanoparticles, especially oxides, as they are considered a scientific pillar thanks to their representation of modern scientific and technological achievements^6,7^. Metal oxide nanoparticles have been widely employed in a variety of applications, such as silver, iron, copper, gold, and oxide nanoparticle Such as iron oxide (hematite α-Fe_2_O_3_^8^, magnetite Fe_3_O_4_^9^), zinc oxide^10,11^, and copper oxide (cuprous oxide Cu_2_O^12^, and cupric oxide CuO^13^), It has been widely used in many different applications in materials engineering, biochemistry, and medicine^14,15^. These nanoparticles, hematite (α-Fe_2_O_3_), zinc oxide, and cupric oxide (CuO) nanoparticles have piqued the interest of researchers because of their unusual structural, optical, and catalytic capabilities, large surface area, and corrosion resistance making them a promising choice for catalysis and biological applications^16^. (α-Fe_2_O_3_, CuO, and ZnO) nanoparticles were reckoned to be efficient and can serve as drug carriers. Also, (α-Fe_2_O_3_, CuO, and ZnO) nanoparticles have a variety of medical applications, such as antibacterial, antifungal, anti-cancer, and anti-diabetic activities.

The inductively coupled plasma mass spectrometry technique was used to demonstrate the adsorption and elimination capacity of heavy metals present in the reservoir water by the various adsorbents: ZnO, α-Fe_2_O_3_ and CuO NPs. In addition, the field water is a gold mine that could contain up to 60% of unexploited crude oil and could be used in various industrial fields^17,18^.

The photocatalytic degradation of oily water (OIW) saturated of hydrocarbon and total suspended solids (TSS) of waste-water associated with crude oil production was studied using several nanoparticles α-Fe_2_O_3_, CuO, and ZnO, prepared by green synthesis. In addition to removing heavy metals. X-ray diffraction (XRD), scanning electron microscope (SEM), and Fourier transforms infrared spectroscopy (FTIR) were used for nanoparticle characterization. These nanoparticles have been found to be very efficient and the total photo mineralization of these organics to carbon dioxide and water occurs in air-equilibrated solution within 1 h.

## Experimental

### Chemicals, reagents, and plant materials

*Portulaca oleracea L.* leaves were collected from El Oued, Southeast Algeria, the experimental research conducted in this study comply with relevant institutional, national, and international guidelines and legislation for research on plant material. Zinc Acetate (Zn (CH_3_COO)_2_· 2H_2_O, 98%), Ferric chloride (FeCl_3_·6H_2_O, 98%), Copper sulfate (CuSO_4_.5H_2_O, 98%) were purchased from Sigma-Aldrich, Germany. Distilled water was used in all the experiments.

### Preparation of plant extract and analysis

First, 250 g fresh and collected *Portulaca oleracea* leaves from local farms in the El-Oued region (southeast of Algeria) were washed with tap water to get rid of dirt and organic deposits on the leaves and then rinsed repeatedly with demineralized water, then crushed and filtered. Fresh leaf components are extracted by mixing 250 g of leaves with 900 ml of distilled water in a 1000 mL glass beaker. The mixture was stirred for 15 min at room temperature. Then filter it using the decanting method and keep the extract cool by storing it at 4 °C for further use^19–21^.

### Green synthesis of α-Fe_2_O_3_, ZnO, and CuO nanoparticles

Modified protocols from previous studies were used for the synthesis of α-Fe_2_O_3_, ZnO, and CuO NPs by a green method using plant extract^22,23^. For the synthesis of α-Fe_2_O_3_, ZnO, and CuO NPs. Different solutions of mineral salts (Ferric chloride (FeCl_3_·6H_2_O), Zinc Acetate (Zn(CH3COO)_2_·2 H_2_O), and copper sulfate (CuSO_4_.5H_2_O)) were prepared using distilled water. About 10 mL of every concentration was mixed with 1 mL of the *Portulaca oleracea* leaves extract with continuous stirring. With controlled and continuous stirring (600 rpm) at 70 °C for 2 h, the color of the solution changes. The precipitate was then centrifuged and washed several times with de-ionized water and dried at 100 °C in an oven for 48 h. Finally, the α-Fe_2_O_3_, ZnO, and CuO NPs were grinded into a fine powder^24,25^ .

The final solid product was collected using centrifugation and washed several times well with distilled water. The product was dried over night at 100 °C then is heated in a furnace at 500 °C for 3 h. The resulting powders were stored in containers for different characterizations^26–28^.

### Characterization of α-Fe_2_O_3_, ZnO, and CuO anoparticles

An all-purpose characterization method called UV–Vis spectroscopy is used to check optical characteristics including transparency and band gap. Shimadzu UV–Vis spectrophotometer model 1800, operating in the 200–900 nm wavelength range, is the instrument. Distilled water was employed as a reference solvent throughout the analysis of the samples in a quartz cell. The existence of functional groups in the chemical extract of the leaves was first ascertained using Fourier transform spectroscopy, and then the composition of α-Fe_2_O_3_, ZnO, and CuO NPs linkages was studied following calcination at 500 °C. The ATR instrument was utilized in the 4000–400 cm^−1^ range. By utilizing an X-ray diffractometer (Rigaku Miniflex 600) with a Cu-K (= 1.5406 ) in the 2 range of 10–90°, while X-ray was produced with 30 kilovolts and at 20 mA, the structure and grain size of α-Fe_2_O_3_, ZnO, and CuO were studied. Scanning electron microscopy (SEM) with energy dispersive X-ray spectroscopy was used to analyze the morphology and form of the nanoparticles (EDAX).

### Purifying petroleum water from hydrocarbons and heavy metals

#### Equipment used

ICP-MS (Inductively Coupled Plasma Mass Spectrometry) the most sensitive method for determining the proportion of metals. It allows simultaneous identification of different ions in the same solution.

ICP-MS product HTDS, Model NexION 2000, it consists essentially of Water-cooled spray chamber, gas mass flow regulator (nebulizer gas, plasma gas and auxiliary gases), nebulizer with variable speed peristaltic pump, valve system and 0.5 mL injection loop, extractor for evacuating impurities, automatic sampler fitted with an injection system, syngetix operating software, 20 mL polypropylene conical tubes with polypropylene caps, Argon and helium gas supply cylinders at 99.9999% purity each, Ultra-pure water, multi-element stock standard solution, each element at a concentration of 1000 mg/litre, Mono-elemental standard solutions, Solutions of the reference element (internal standard) or optimization solution for the standard mode and for the KED mode (Kinetic energy discrimination).

*Adsorbents* Particles of iron oxide (α-Fe_2_O_3_), copper oxide (CuO) and zinc oxide (ZnO) synthesized by the green method.

TD-500D Oil in Water Meter the analyzer (Revision: C, P/N 100,668) from the United States of America uses UV fluorescence to determine the oil content of oily water comprising crude oil or gas condensates.

An oil in water analyzer (TD-500, Turner Designs Hydrocarbon Instruments, USA) was used to determine the amount of oil or OIW in the generated or separated water.

1 $$ {\mathbf{Oil}} {\mathbf{removal}} \,{\mathbf{efficiency}} \left( \% \right) = \frac{{\left( {{\varvec{C}}_{{\varvec{i}}} - {\varvec{C}}_{{\varvec{t}}} } \right)}}{{{\varvec{C}}_{{\varvec{i}}} }} \times 100 $$

*UV–Visible spectrophotometer* Suspended solids (SS) represent all of the insoluble residues visible to the naked eye and suspended in the water. These materials can be analyzed by a UV–Visible spectrophotometer (brand mat-lib DR6000)—A test sample of 10 mL of petroleum water.

Oily water discharged by the separation units of crude oil extracted from the oil fields of the HASSI MESSAOUD area located in the south of Algeria (31.201183,5.740473).

#### *Sorption study of metal ions on α-Fe*_*2*_*O*_*3*_*, CuO and ZnO NPs*

Sorption studies were performed for eleven different metal As, Be, Cd, Cr, Mn, Mo, Ni, Pb, Sb, Se and Zn. All metal ions were taken in their nitrate form so that the effect of counter ion will be same^29^. To study the loading capacity of these metal ions on α-Fe_2_O_3_ CuO and ZnO NPs, 20 mg of NPs was taken and 10 mL oily water. Solution of different concentrations of metal ions were added. The solution was sonicated for 30 min followed by separation using a magnet and supernatant was analysed for metal ions concentration using ICPMS. A schematic diagram of the study is shown in Fig. 8. The results are obtained directly using the software integrated into the ICP device (Syngetix operating software.), they are calculated in mg/L using a linear regression of the response of the standards and are corrected by internal standards. If necessary, the data should be reintegrated following the software program. If applicable, multiply by the dilution factor.

#### Analysis of an oily in water sample (OIW)

A standard sample is used to calibrate the TD-500 m before analyzing a sample. As a first step, standard samples of known oil content are prepared. We put 100 mL of water in a bottle to analyze and adjust the pH at a value of < 2 with HCl (4 or 5 drops). After adding 10 mL of solvent (hexane) and shaking vigorously for 2 min, the hydrocarbons in the water were extracted. Let settle for about 10 min and fill with a syringe 3/4 of a bowl with the test socket extracted from the ampoule and wipe it clean. Place the bowl in the device and read the response value. The analyzer responds within 5 s. Note the concentration of hydrocarbons in ppm^30,31^.

#### Total suspended solids (TSS)

A volume of 10 mL of petroleum water is poured into a transparent tank to allow the light beam to pass through the sample. The cuvette is then placed in the UV–VISIBLE spectrophotometer. The analysis is started. After 10 min the reading is made on the screen of the device^32,33^.

## Results and discussion

### Crystal structure and composition

*Portulaca oleracea*, commonly known as garden purslane, is referred to by the Arabic name "*Redjila*". The *Portulaca* genus includes approximately 40 species, mainly tropical and adapted to warm climates, including *Portulaca oleracea L*.

The leaves of *Portulaca oleracea* contain: alpha-linoleic acid, alpha-tocopherol and ascorbic acid with a very large amount than the leaves of spinach^34^.

**Figure 1:**
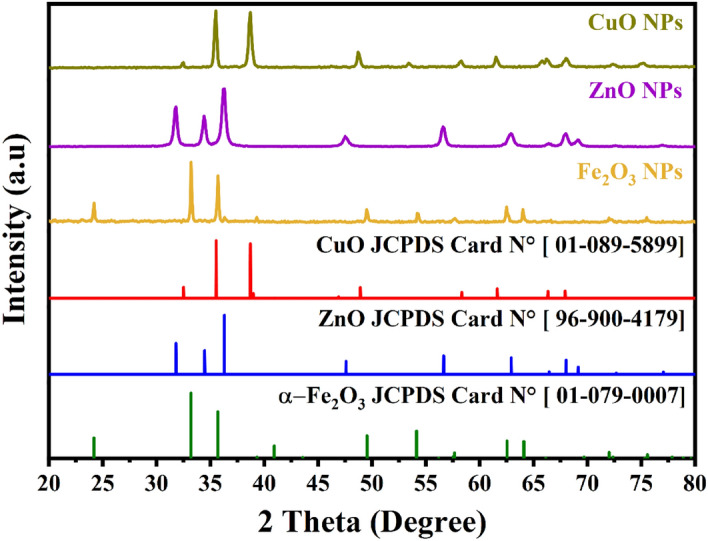
X-ray diffraction patterns of the α-Fe_2_O_3_, CuO and ZnO nanoparticles.

The Fig. 1 exhibits typical XRD patterns of synthesized and annealed at 500 °C *α-Fe*_*2*_*O*_*3*_*, CuO and ZnO* nanoparticles, the sample of *α-Fe*_*2*_*O*_*3*_ shows very thin peaks, indicating the fine nature and small crystallite size of the particles . Show in Fig. 1 the spectrum indicate the presence of Hematite α-Fe_2_O_3_. As depicted by the peaks at 2θ values of 24.13°, 33.15°, 35.45°, 40.70°, 49.47°, 54.04°, 62.90° and 63.98° which correspond to the crystal planes of (012), (104), (110), (113), (024), (116) , (214) and (300) of Hematite a-Fe_2_O_3_ phase^35^ .All the reflection peaks are matching well with the expected to rhombohedral structure of α-Fe_2_O_3_ (space group: R-3c), respectively. Those planes accord well with the (JCPDS Card N^°^. 01–079-0007).

Figure 1 illustrates the XRD pattern of the cupric oxide (CuO) NPs prepared using *Portulaca oleracea L*. extract. The presence of cupric oxide crystals is confirmed by this diffractogram (CuO). Peaks position with 2θ values of 32.5°, 35.5°, 38.8°, 48.8°, 58.3°, 61.7°, 66.3°, and 68.4° correspond to the crystalline planes of (110), (002), (111), (202), (202), (113), (311), and (220), which support the creation of the monoclinic crystal structure for CuO Card N°. (JCPDS-01–089-5899)^36^.

As can be seen from the XRD patterns in Fig. 1, the sample displayed a similar peak position with 2θ values of 31.8°, 34.46°, 36.29°, 47.59°, 56.65°, 62.93°, 66.45°, 68.02°, 69.14°, 72.68°, 77.05° which have been credited to the crystal planes of (100), (002), (101), (012), (110), (013), (200), (112), (201), (004), and (011) respectively where ZnO NPs having a hexagonal crystal structure (Space group P 63 m c (186) and lattice parameters of a = 3.24940 Å c = 5.20380 Å) JCDPS Card N°. (96–900-4179)^37^. Crystallite diameters in the various samples ranged from 20.12 nm to 25.04 nm (Table 1).

**Table 1:** Crystallite size of α-Fe_2_O_3_, CuO and ZnO NPs obtained by different pH values.

Samples	Crystallite size (nm)	Lattice	Lattice parameters	Space group	References
α-Fe_2_O_3_	20.12	Rhombohedral	a = 5.0285 Å c = 13.7360 Å	R-3c (167)	^35^
CuO	22.25	Monoclinic	a = 4.6890 Å b = 3.4200 Å c = 5.1300 Å	Cc (9)	^36^
ZnO	25.04	hexagonal	a = 3.24940 Å c = 5.20380 Å	P 63 m c (186)	^37^

### FTIR spectroscopy analysis

To determine the potential phytochemical compounds responsible for the green synthesis of the nanoparticles, FT-IR analysis was employed on the synthesized powder of *α-*Fe_2_O_3_, CuO, and ZnO nanoparticles as well as the extract of *Portulaca oleracea L*. leaf.

In this study, bands in the IR spectra that are indicative of the functional groups of biomolecules involved in the creation and stability of nanoparticles were detected.

**Figure 2:**
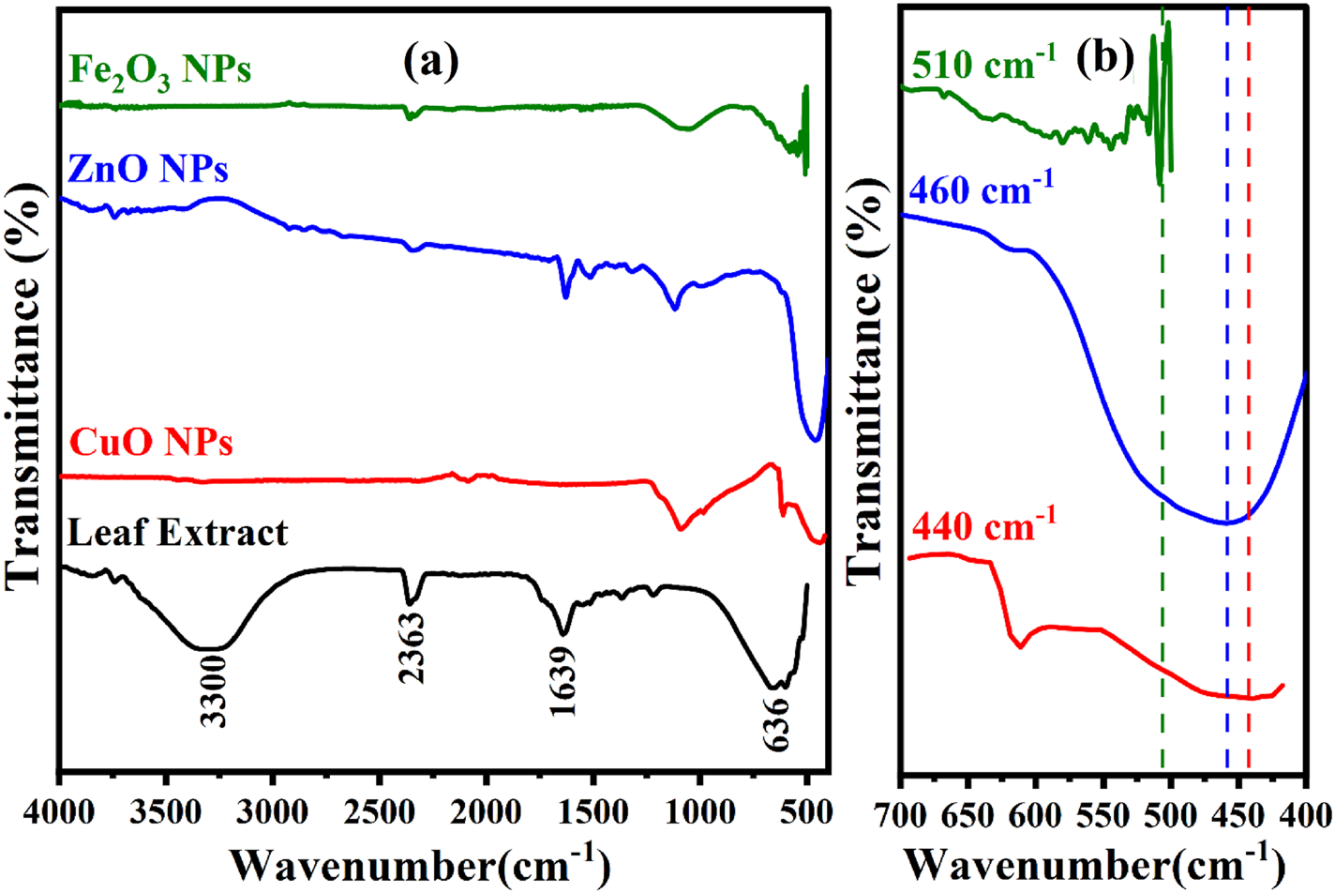
(**a**) FTIR spectra of Portulaca oleracea L. leaf Extract, α-Fe_2_O_3_, CuO, and ZnO NPs; (**b**). Zoomed view range (400 to 700 cm^−1^) of synthesized α-Fe_2_O_3_, CuO, and ZnO NPs.

Figure 2 presents the FT-IR spectrum of a *Portulaca oleracea L*. leaf extract together with the synthesized spectra of α-Fe_2_O_3_, CuO, and ZnO nanoparticles. The FT-IR spectra of *Portulaca oleracea L* leaf extract is shown in Fig. 2 (a). This spectrum showed some peaks at 3300, 2363, 1639, and 636 cm^−1^. The O–H group stretching vibration is what causes the broad band to vibrate at 3300 cm^−1^ ^38^. The band at 2336 cm^−1^ are the typical C–C stretching peaks. Peaks at 1639 cm^−1^ are associated with the stretching vibrations of the aromatics cycles C=C, CC, and CO^39^. Another band at 636 cm^−1^ can be attributed to C–O–H stretching of primary alcohols and C–H out of plane aromatic band^40^.

By comparing the spectrum of the as α-Fe_2_O_3_, CuO, and ZnO nanoparticles each other, Fig. 2(b) shows a peek at 510, 460, and 440 cm^−1^, which, corresponding to the Fe–O, Cu–O, and Zn–O stretching band of α-Fe_2_O_3_, CuO, and ZnO NPs, respectively .

Also a disappearance of the absorbance bands 3300, 2363, 1639, and 636 cm^−1^ associated of phenolic compounds after synthesing α-Fe_2_O_3_, CuO, and ZnO NPs, leads us to proclaim that the leaves extract of *Portulaca oleracea L.* contains phytochemicals such as alcohols, aldehydes, alkanes, and epoxy groups or ether groups which can be responsible for the nucleation process to reduce precursor from M^+^ to M^0^.

### Morphological investigation

**Figure 3:**
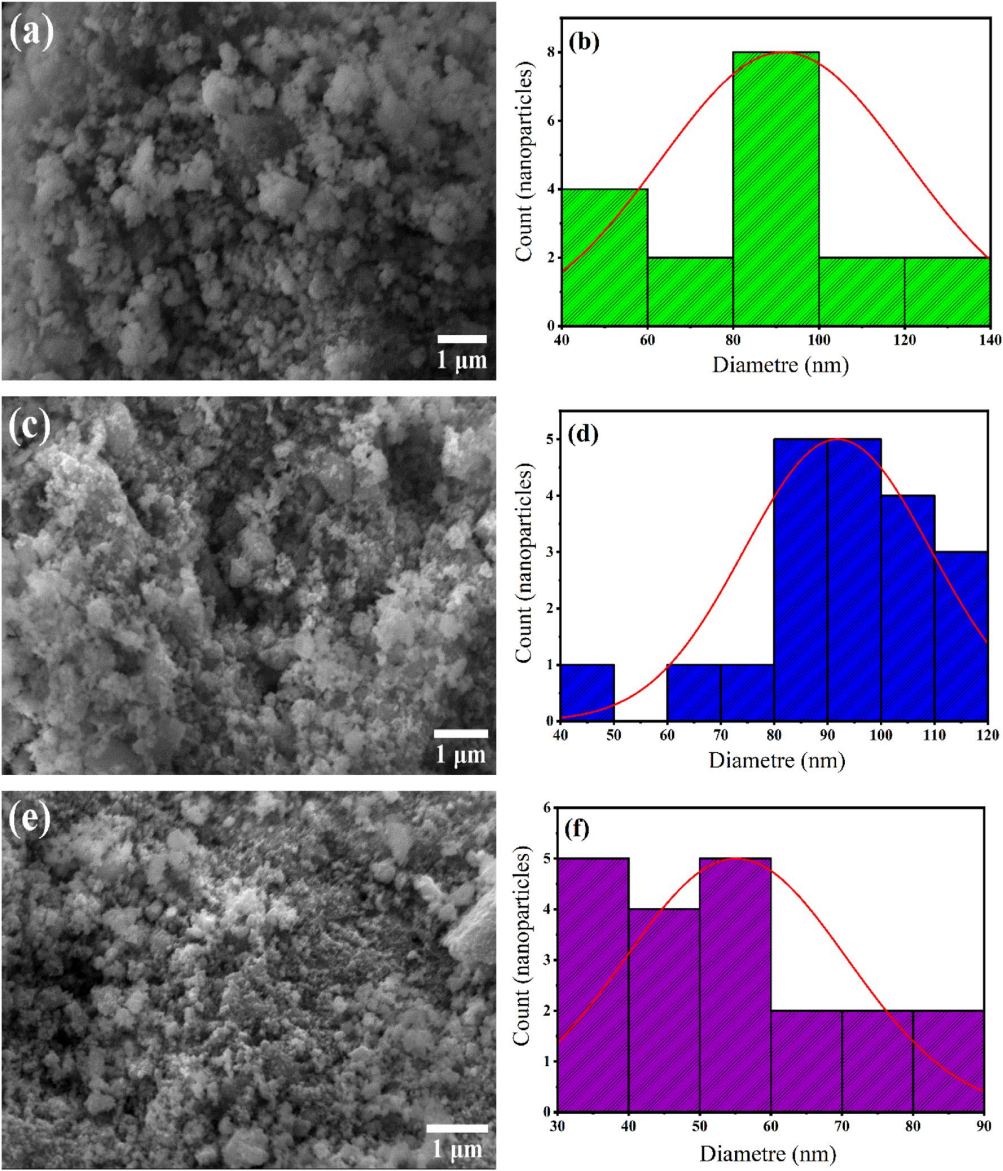
SEM images and particle size distributions of green synthesized α-Fe_2_O_3_, CuO, and ZnO NPs: (**a**, **b**) α-Fe_2_O_3_, (**c**, **d**) CuO, and (**e**, **f**) pH = ZnO).

The formation of α-Fe_2_O_3_, CuO, and ZnO NPs and their morphological dimensions were investigated using the SEM. The SEM images of the produced α-Fe_2_O_3_, CuO, and ZnO NPs are shown in Fig. 3 (a, c, e). Nearly all of them are found to be spherical or oval in shape. With a few widely scattered solitary particles. The particle size distribution histograms given in Fig. 3 (b, d, f) indicate that the average size distribution of biosynthesized α-Fe_2_O_3_, CuO, and ZnO NPs is largely around 80 nm. We can determine that the gathered particles are crystals by comparing their size to that of crystals.

**Figure 4:**
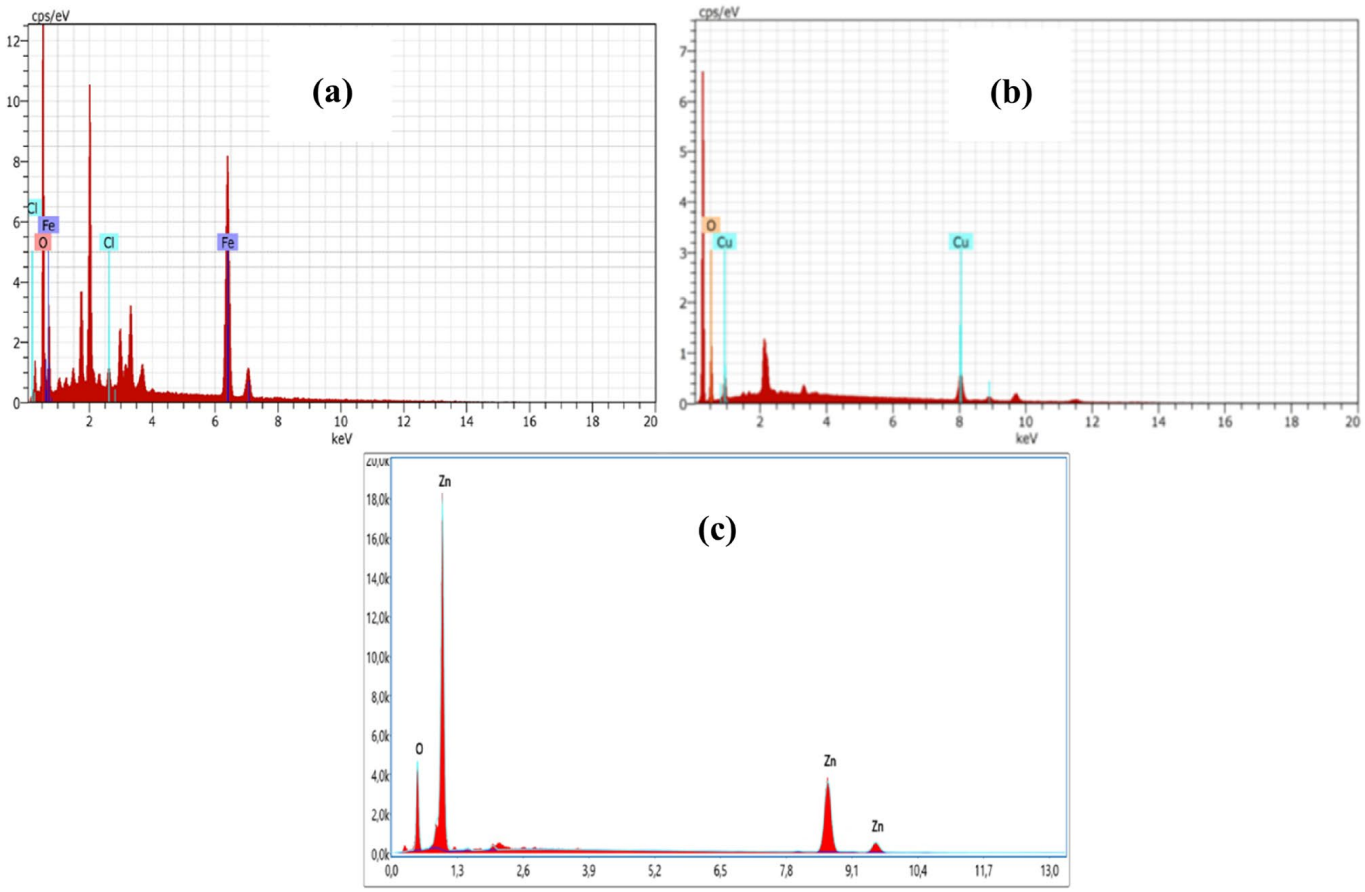
EDS of (**a**) α-Fe_2_O_3_,(**b**) CuO,(**c**) and ZnO Nanoparticles.

The energy dispersive X-ray analysis (EDAX) of α-Fe_2_O_3_, CuO, and ZnO nanoparticles is displayed in Fig. 4. The EDAX spectra of α-Fe_2_O_3_ nanoparticles is shown in Fig. 4(a), and the peaks correspond to Fe and O. The paste and grid utilized for the EDAX analysis are where the components Cl in the figure come from. Figure 4 (b,c) The EDAX analysis showed only obvious peaks for the elements Fr, Zn, and O; no other peaks could be found, indicating that the powder was manufactured without any impurities for the CuO and ZnO NPs sample.

Table 2 displays the weight, atomic, and error % percentages of nanoparticles of α-Fe_2_O_3_, CuO, and ZnO. This demonstrates that the Fe:O, Cu:O, and Zn:O ratios in synthetic nanoparticles are not stoichiometric or 1:1. CuO, ZnO, and α-Fe_2_O_3_ NPs.

**Table 2:** Quantitative analysis of α-Fe_2_O_3_, CuO and ZnO NPs using EDX.

	Element	Weight %	Atomic %	Error %
α-Fe_2_O_3_	Fe K	28.39	60.13	0.79
	O K	18.12	38.38	2.31
	Cl K	0.7	1.19	0.06
CuO	Cu K	72.39	39.76	0.27
	O K	27.61	60.24	0.53
ZnO	O K	18.56	48.21	8.37
	Zn K	81.44	51.79	2.26

### Bandgap and Optical characteristics

**Figure 5:**
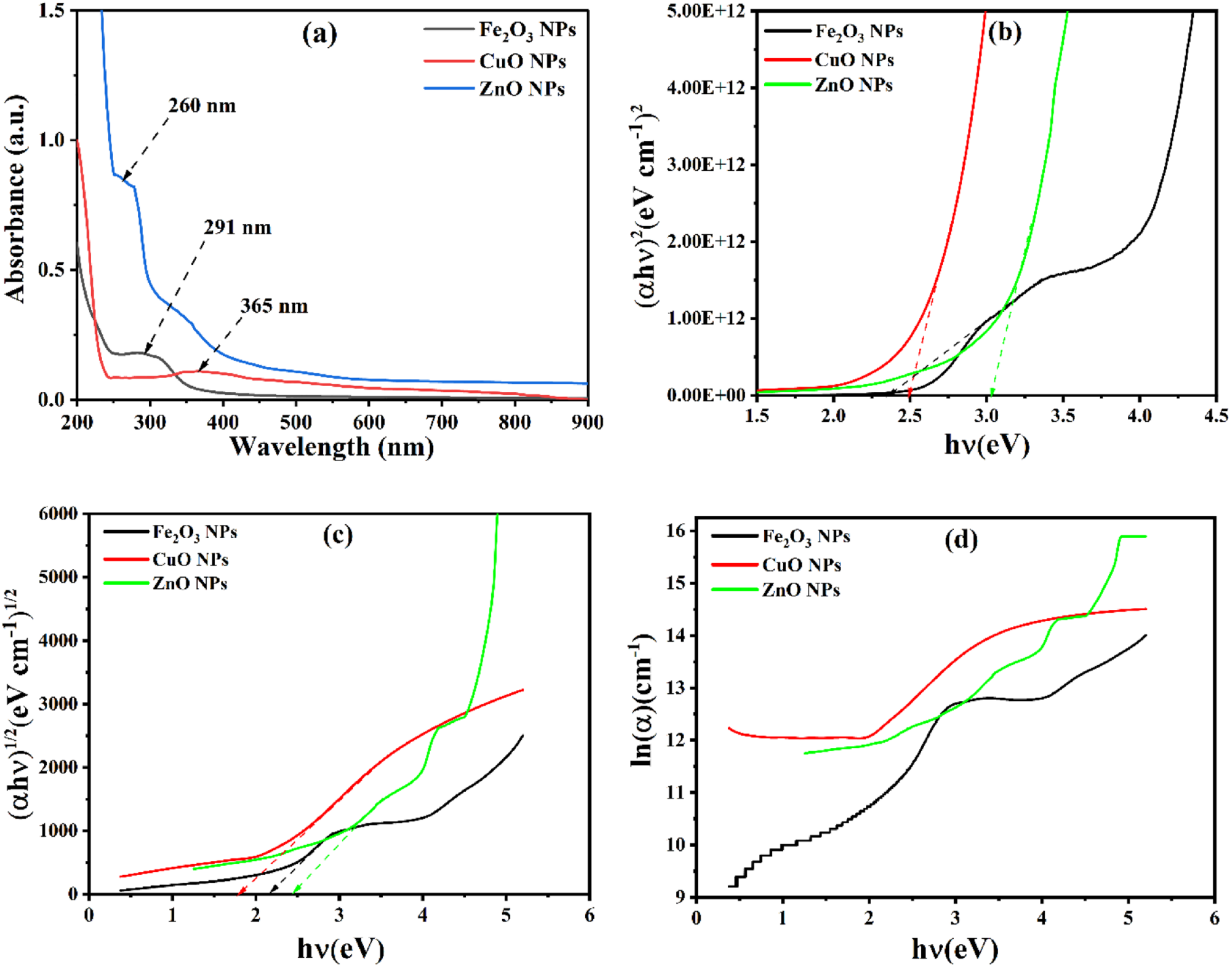
Optical properties of the α-Fe_2_O_3_, CuO, and ZnO NPs: UV–vis spectra (**a**); optical bandgap energy (**b**) ; indirect bandgap energy (**c**) transitions relying on Tauc’s method; (**d**) Urbach energy.

The UV–Vis spectra of α-Fe_2_O_3_, CuO, and ZnO nanoparticles synthesized using a *Portulaca oleracea L*. Leaf extract are shown in Fig. 5(a). As can be seen from this figure, one peaks of maximum absorption are exhibited for every nanoparticle. The absorption peak at 291, 365, 260 nm gives a clue that α-Fe_2_O_3_, CuO, and ZnO nanoparticles, respectively.α-Fe_2_O_3_, CuO, and ZnO nanoparticles' estimated optical band gap (Eg) can be calculated by extrapolating from the absorption edge, which is provided by Tauc's relation^41,42^:

2 $$ \left( {\alpha hv} \right) = A\left( {hv - E_{g}^{opt} } \right)^{n} $$

where $h$ is the Planck constant, $A$ is a constant, $hv$ is the energy of light, n is a factor is equal to 1/2 or 2 for direct and indirect transition, and α is the absorption coefficient. Plotting $\left( {\alpha h\nu } \right)^{2}$ and $\left( {\alpha h\nu } \right)^{1/2}$ vs photon energy allows one to determine the optical bandgap energy for direct ${\text{E}}_{{{\text{g}}1}}^{{{\text{opt}}}}$ and indirect $E_{g2}^{opt}$ transitions (hv). The value of $E_{g}^{opt}$ is calculated by extrapolating to $\left( {\alpha h\nu } \right)^{2} = 0$ for direct transition and $\left( {\alpha h\nu } \right)^{1/2} = 0$ for indirect transition in NPs of α-Fe_2_O_3_, CuO, and ZnO. The plot of $\left( {\alpha h\nu } \right)^{2}$ and $\left( {\alpha h\nu } \right)^{1/2}$ versus $hv$ ^23^ is shown in Fig. 5 (b–c). The energy gap was calculated from the point where the energy axis and the edge of the linear portion of absorption met. The photon energy is equal to $E_{g}^{opt}$ when $\left( {\alpha h\nu } \right)^{n}$ is zero. Table 3 shows the samples' optical band gaps.

**Table 3:** Direct, indirect optical band gaps, and Urbach energies of synthesized α-Fe_2_O_3_, CuO, and ZnO NPs.

Samples NPs	Direct optical bandgap (eV)	Indirect optical bandgap (eV)	Urbach energy (eV)
α-Fe_2_O_3_	2.36	2.16	0.890
CuO	2.49	1.79	0.689
ZnO	3.02	2.45	0.312

The band tail energy, sometimes called Urbach energy $E_{u}$, can be seen in UV–vis spectra. It has been found that the Urbach energy $E_{u}$ values decrease with crystal size in the case of α-Fe_2_O_3_, CuO, and ZnO NPs forms, leading to a decrease in crystallinity and structural disorder. Utilizing the reciprocal values of the slopes of the linear component of the $\ln (a)$ vs photon energy $h\nu$, the Urbach energy $E_{u}$ is computed (Fig. 5d). According to Urbach (1953)^43^, the absorption coefficient and photon energy have an exponential relationship close to the band edge. Table 3 displays the calculated Urbach energy values for the samples.

3 $$ \ln {\text{a}} = { }\frac{{{\text{hv}}}}{{{\text{E}}_{{\text{u}}} }} + {\text{ constant }}(\ln {\text{a}}_{0} ) $$

The reciprocal of the slope of the linear fit section of the curve's photon energy was used to determine the Urbach energy $E_{u}$. The latter is based on the energy difference between the ends of the valence and conduction band tails; the lower the energy, the less turbulence; however, the disorder may change depending on the presence of modifying oxides.

### Removal of heavy metals

**Figure 6:**
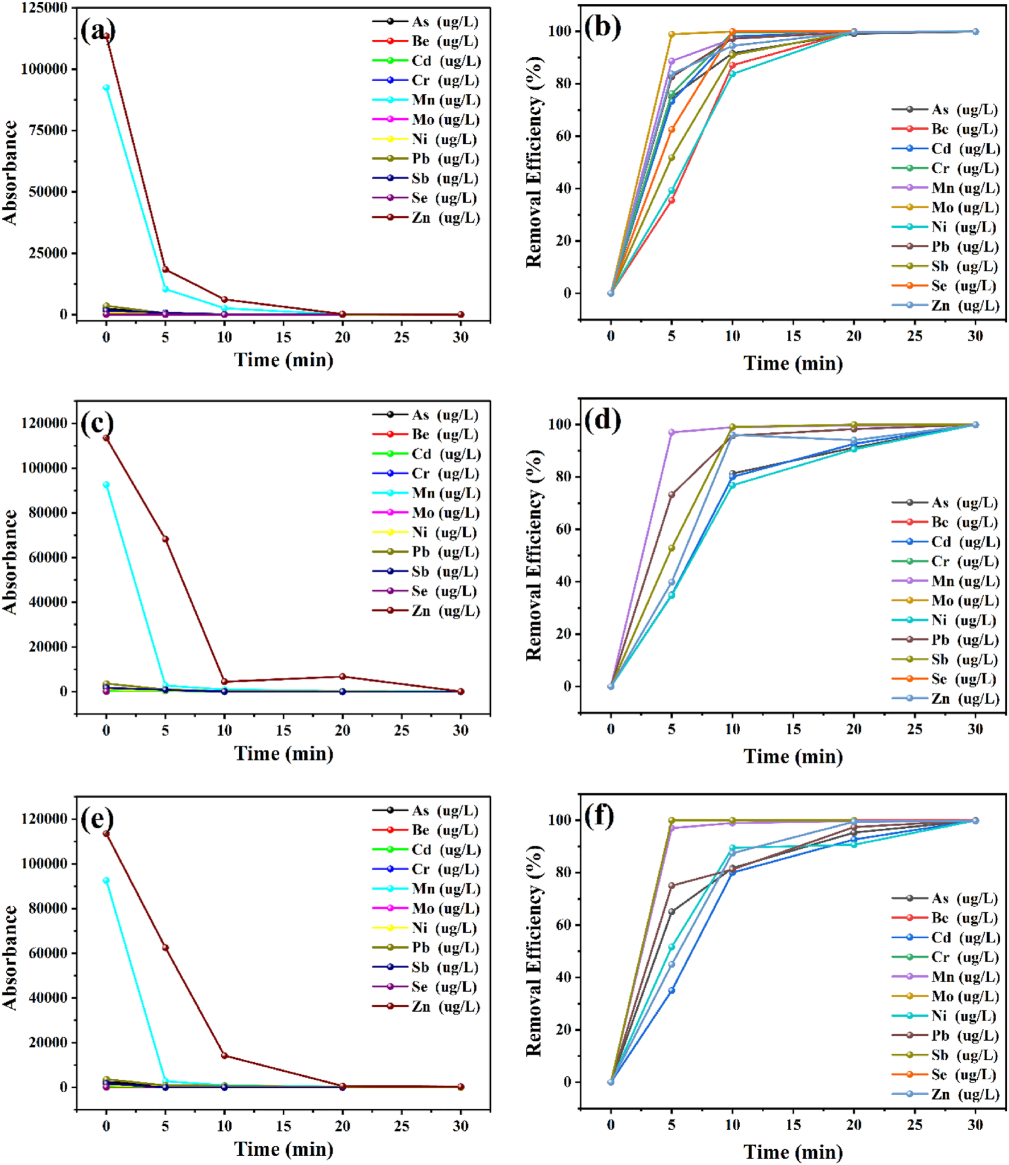
Time effect and efficiency rate of (**a**, **b**) α-Fe_2_O_3_, (**c**, **d**) CuO, and (**e**, **f**) ZnO for removal of heavy metals from the aqueous phase.

The rate of extraction of As(III), Be(II), Cd(II), Cr(VI), Mn(II), Mo(II), Ni(II), Pb(II), Sb(III), Se(-II), and Zn(II) by α-Fe_2_O_3_, CuO and ZnO NPs was studied by equilibrating the 20 g nanoparticles with a series of ion solutions for different time intervals. Figure 6 shows the amount of As, Be, Cd, Cr, Mn, Mo, Ni, Pb, Sb, Se, and Zn (Table 4) adsorbed at a contact time t (min). The results have indicated that ~ 80% of the adsorbed of heavy metals takes place in 5 and 10 min respectively, and the time required for both ions to reach a Mineralization percentage of 99.99% is less than 30 min shown in Fig. 6.

**Table 4:** Results of analysis by ICP-MS of the reservoir oily water (OIW) before treatment and after.

OIW after treatment	As 75 = (ug/L)	Be 9 (ug/L)	Cd 111 (ug/L)	Cr 52 (ug/L)	Mn 55 (ug/L)	Mo 98(ug/L)	Ni 60 (ug/L)	Pb 208 (ug/L)	Sb 121 (ug/L)	Se 82 (ug/L)	Zn 66 (ug/L)
α-Fe_2_O_3_	1,370	0.000	0,406	0.000	71,728	0.000	0,647	2,998	0.000	0.000	98,867
CuO	1,212	0.000	0,355	0.000	62,802	0.000	0,428	2,399	0,000	0.000	87,992
ZnO	1,310	0,001	0,392	0.000	69,370	0.000	0,669	0,935	0.000	0.000	327,605

Qtit metals	As 75 (mg/L)	Be 9 (mg/L)	Cd 111 (mg/L)	Cr 52 (mg/L)	Mn 55 (mg/L)	Mo 98 (mg/L)	Ni 60 (mg/L)	Pb 208 (mg/L)	Se 82 (mg/L)	Sn 118 (mg/L)	Zn 66 (mg/L)
OIW before treatment	2,473	0,062	0,546	0,349	92,547	0,206	1,009	3,601	1,706	0,160	113,481

**Figure 7:**
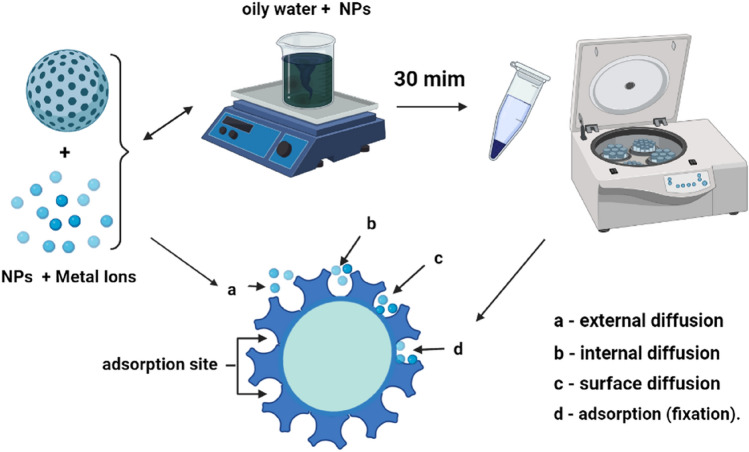
Schematic of the experiment and Mechanism of the transfer of an adsorbate to the adsorption site within a grain.

The adsorption behaviour described above can be explained on the basis of surface charge and proton competitive sorption. The increase in deprotonation induces the increase of negatively charged sites, which enhances attractive electrostatic interaction and ion exchange between the NPs sorbent surface and metal ions and consequently leads to the enhancement of the adsorption capacity. As shown in Fig. 7,

The knowledge of the parameters of the adsorption equilibrium makes it possible to deduce the adsorption capacities of a support. The determination of the kinetic parameters must, in addition, be the determination of the kinetic parameters must, in addition, be carried out for the prediction of the curves. The transfer of an adsorbate from the transfer of an adsorbate from the liquid phase to an adsorption site, represented by Fig. 7, involves the following steps:

*1st step* (the external diffusion): the transfer of solute molecules from the external liquid phase to the liquid phase bound to the adsorption site to the liquid phase bound to the solid particle (by diffusion and convection).

*2nd step* (the internal diffusion): the transfer of the solute through the liquid film towards the external surface of the adsorbent.

*3rd step* the diffusion of the adsorbate inside the particle of the adsorbent under the effect of the concentration gradient. The adsorbate molecule can diffuse from one adsorption site to another other either in the Free State (after desorption) in the intraparticle liquid phase (migration characterized by a diffusion coefficient Df), or in the adsorbed state, from an adsorption site towards an adjacent site (migration of surface characterized by a coefficient of diffusion Ds).

*4th step* adsorption (fixation).

The results obtained (Table 4) show that half an hour of stirring leads to a decrease in the concentration of all the metals present in the sample. By going from 10 min of agitation to 30 min, Note that by going from 5 to 30 min of agitation, we were able to completely get rid of most of the elements present in petroleum water percentages varying between 90 and 100% (see Fig. 6), thanks to the efficiency of the nanoparticles used to filter this oily water. This is due to the high quality of the specific surface of the absorbent material.

### Photocatalytic degradation of hydrocarbon in oily water (OIW) and remove Total Suspended Solids (TSS)

The photocatalytic process is an alternative and cutting-edge technique increasingly used to treat produced water. In this study, petroleum was used as a produced water indicator.

**Figure 8:**
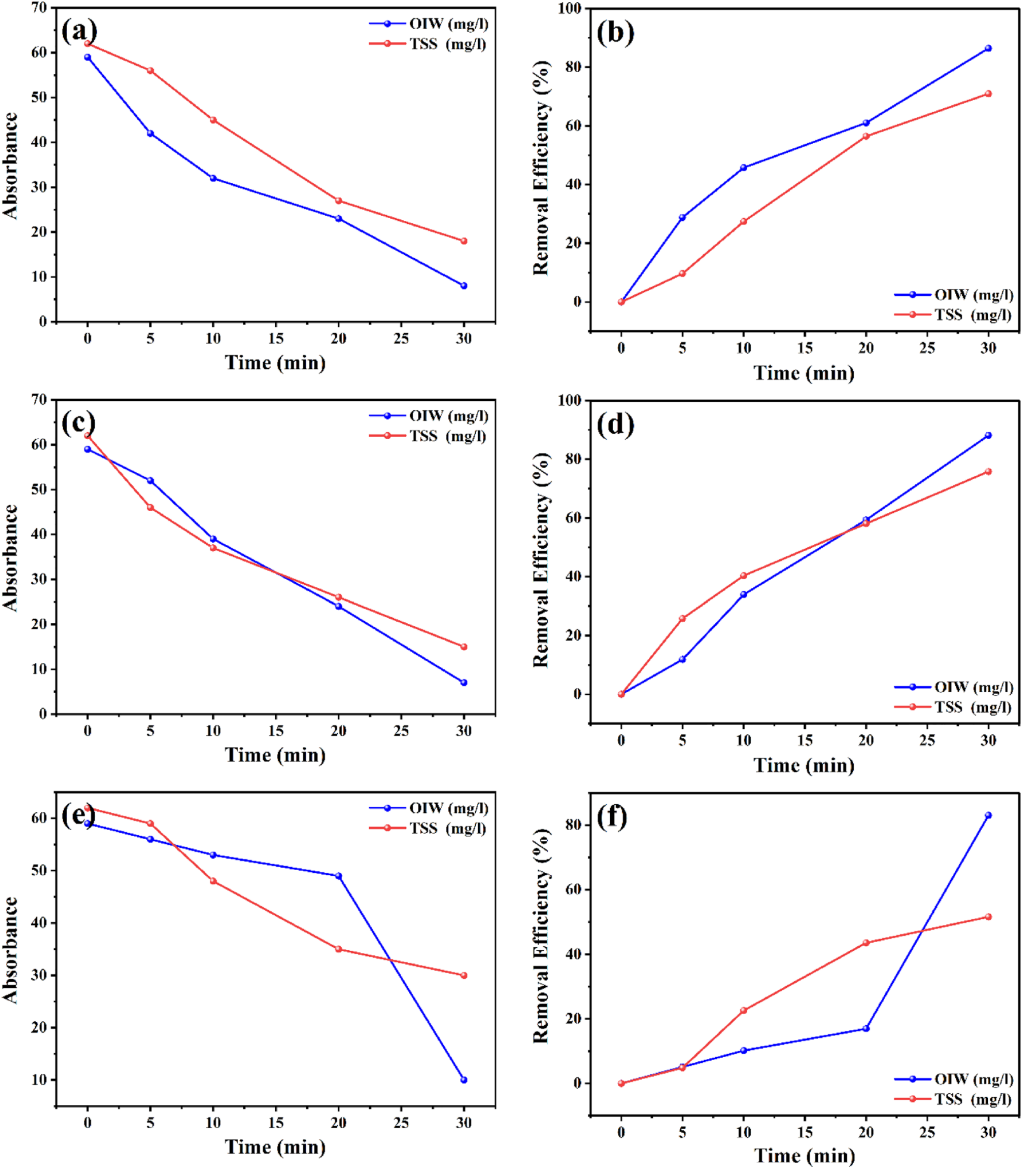
The photo-degradation rate hydrocarbon in oily water (OIW) and removing of Total Suspended Solids (TSS).

In Fig. 8 we discovered that 80% of the hydrocarbon degradation for photocatalyst NPS uses a UV sunlight source. The outcome also demonstrates that OIW deterioration was more pronounced when reactions for 30 min under visible light. Removing the (TSS) in the oily water by 70% as shown in Fig. 8, due to the quality of the surfaces of the three studied nanoparticles.

**Figure 9:**
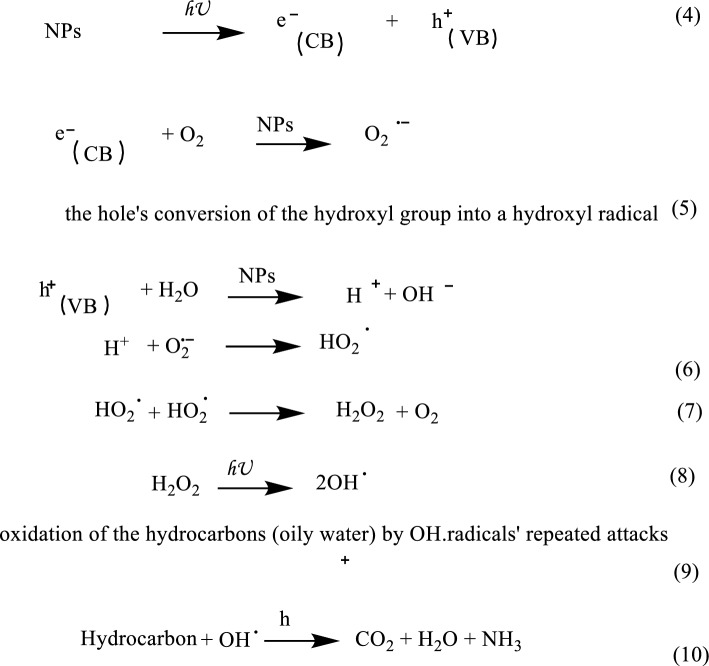
illustrates the predicted reaction process for the photocatalytic breakdown of the pollutant's hydrocarbon.

In the photocatalytic process, nanoparticles transform organic contaminants into CO_2_, H_2_O, and reactive oxidizing species like oxygen or air^44^. Nanoparticles are used in a wide range of industries^45–52^. When a photon with energy (hv) equal to or greater than the band gap of semiconductor photocatalytic nanoparticles is absorbed, the mechanism of the photocatalytic process is said to have begun^53^. Thus, positive holes (h^+^) and electrons (e^−^) are created on the surface of nanophotocatalysis as a result of electrons being transferred by photoabsorption from the valence band to the conduction band^54^. As a result, the interactions between positive holes in CB and water produce hydroxyl radicals (OH) Fig. 9.

**Figure 10:**
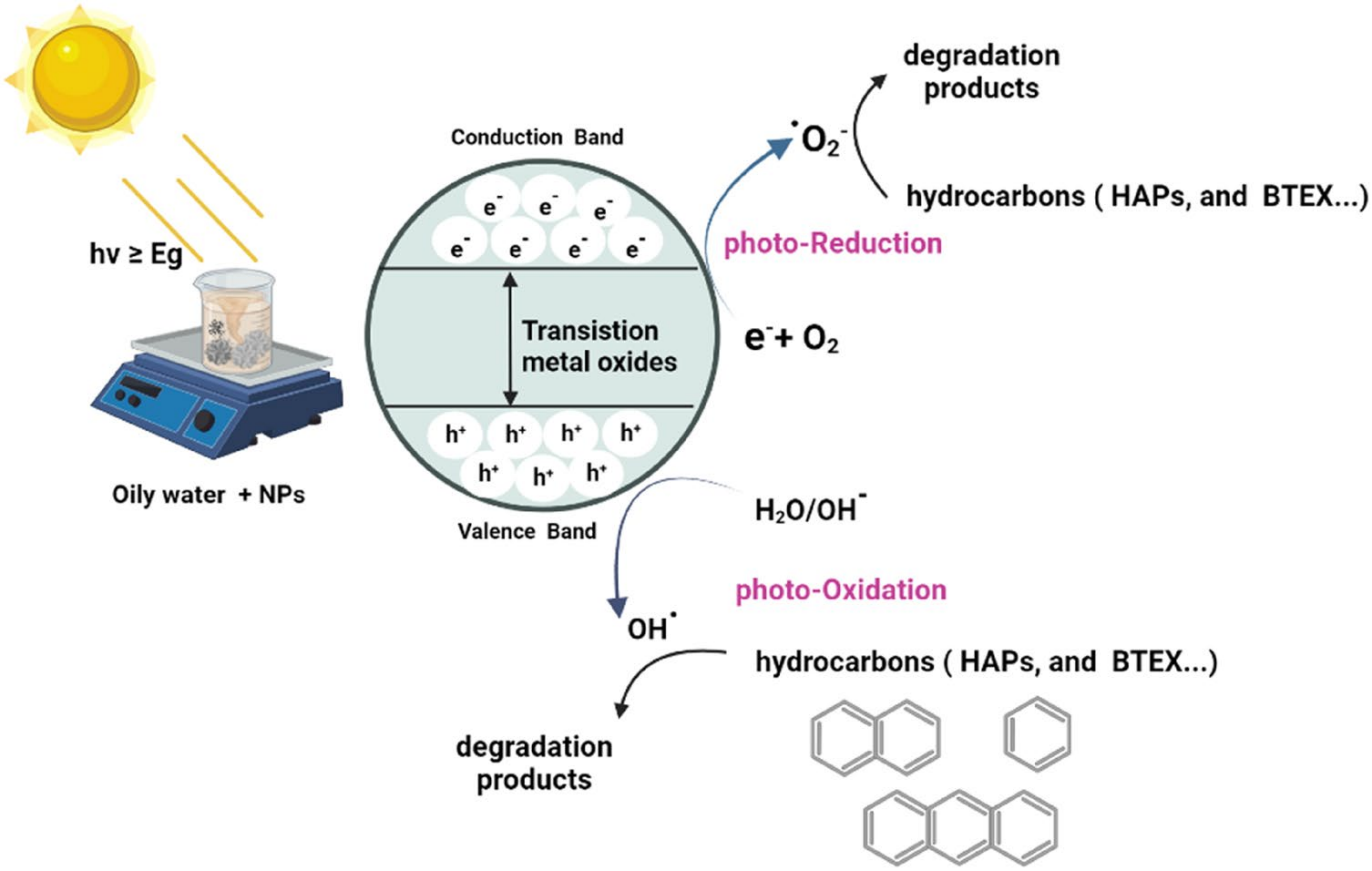
Mineralization mechanism by photodegradation of hydrocarbons (oily water) using nanoparticles.

Based on the previous discussions, Fig. 10 discusses a potential reaction pathway using the hydroxyl and superoxide radicals for the potential creation of intermediates. The primary oxidation step is thought to be started by hydroxy radicals, which are produced after photogenerated holes trapped at the α-Fe_2_O_3_, CuO, and ZnO NPs surface oxidize hydroxyl ions.

## Conclusion

The porous nanoparticles were utilized for the degradation of hydrocarbon in oily water (OIW) and remove Total Suspended Solids (TSS) and the total adsorption of heavy metals (Bi, Cr, Mo, Sb and Se). In addition about 80% of the hydrocarbon degradation for photocatalyst NPS uses a UV sunlight source. Mainly polyhydroxylated and carbonylated intermediates are formed, which are ultimately converted to carbon dioxide. The amount of CO as the final mineralization product. And it has proven that it can remove heavy metals and purify water from all suspended impurities, the potentiality exists for the application of photocatalytic methods in the purification of water polluted by hydrocarbons.

## Data Availability

All data generated or analysed during this study are included in this published article.

## Abbreviations

NPs: Nanoparticles
ZnO: Zinc oxide
CuO: Cupric oxide
α-Fe_2_O_3_: Hematite
*OIW*: Oily in water
*TSS*: Total suspended solids
