# Author Correction: Removal of hydrocarbons and heavy metals from petroleum water by modern green nanotechnology methods

**DOI:** 10.1038/s41598-025-94460-w

**Published:** 2025-04-08

**Authors:** Abderrhmane Bouafia, Souhaila Meneceur, Souheyla Chami, Salah Eddine Laouini, Henda Daoudi, Souheila Legmairi, Hamdi Ali Mohammed Mohammed, Narimene Aoun, Farid Menaa

**Affiliations:** 1 Department of Process Engineering, Faculty of Technology, University of El Oued, 39000 El-Oued, Algeria; 2 Laboratory of Biotechnology Biomaterial and Condensed Matter, Faculty of Technology, University of El Oued, 39000 El-Oued, Algeria; 3 Laboratory of Polymers Treatment & Forming, Faculty of Technology, M’Hamed Bougara University, 35000 Boumerdes, Algeria; 4 Laboratory of Bioresources, Integrative Biology and Exploiting, Biotechnology Higher Institute, Monastir University, 5000 Monastir, Tunisia; 5 Department of Chemistry, Faculty of Exact Sciences and Informatics, University of Jijel, 18000 Jijel, Algeria; 6 Department of Nanomedicine and Advanced Technologies, CIC-Fluorotronics, Inc, San Diego, CA 92037 USA

Correction to: *Scientific Reports* 10.1038/s41598-023-32938-1 Published online 06 April 2023.

The original version of this Article contained an error in the Results and discussion section, under the subheading ‘Crystal structure and composition’, where a passage in French was inadvertently included.

“*Portulaca oleracea* communément appelée pourpier potager, elle est connue sous le nom arabe "*Redjila*". Le genre *Portulaca* comprend environ 40 espèces de tropicales et des espèces de climat chaud *Portulaca oleracea L*.”

now reads:

*"Portulaca oleracea,* commonly known as garden purslane, is referred to by the Arabic name *'Redjila.'* The *Portulaca* genus includes approximately 40 species, mainly tropical and adapted to warm climates, including *Portulaca oleracea L."*

The original Article has been corrected.

